# Cutoff scores for the “Interest game”, an application for the assessment of diminished interest in neurocognitive disorders

**DOI:** 10.3389/fpsyt.2023.1126479

**Published:** 2023-03-20

**Authors:** Valeria Manera, Roxane Fabre, Lyne Daumas, Radia Zeghari, Alexandre Derreumaux, Magali Payne, Justine Lemaire, Guillaume Sacco, Auriane Gros, Philippe Robert

**Affiliations:** ^1^Université Côte d’Azur, CobTeK, Nice, France; ^2^Association Innovation Alzheimer, Nice, France; ^3^Université Côte d’Azur, Department of Speech Therapy (Departement d’Orthophonie, DON), Nice, France; ^4^Université Cote d’Azur, Nice University Hospital (CHU), Public Health Department, Nice, France; ^5^Université Côte d’Azur, LAMHESS, Nice, France; ^6^Centre Hospitalier Universitaire-Lenval, Service Universitaire de Psychiatrie de l’Enfant et de l’Adolescent, Hôpitaux Pédiatriques de Nice, Nice, France; ^7^Université Côte d’Azur, Centre Hospitalier Universitaire de Nice, Clinique Gériatrique de Soins Ambulatoires, Centre Mémoire de Ressources et de Recherche, Nice, France

**Keywords:** apathy, diminished interest, assessment, application, neurocognitive disorders

## Abstract

Diminished interest is a core feature of apathy that shows high prevalence in people with Mild and Major Neurocognitive disorders (NCD). In the clinical setting, apathy is mainly assessed using clinical scales and questionnaires, but new technologies are starting to be employed to complement classical instruments. Here, we explored the performance of the “Interest game,” a ludic application that assesses personal interests, in discriminating between persons with and without diminished interest based on the Apathy Diagnostic Criteria. Two hundred and twenty-seven elderly participants (56 healthy controls, 118 persons with mild-NCD, and 53 with major-NCD) completed the Interest game and were assessed by clinicians concerning the presence and the severity of apathy. Results showed that the application scores varied with the presence of apathy, the type of disorder, and the education level. Cutoff scores calculated for persons with Mild-NCD resulted in a sensitivity of 0.68 and a specificity of 0.65 for the main score index, suggesting the interest of employing this application in the clinical setting to complement the classical assessment.

## Introduction

Neurocognitive disorders (NCD) are characterized by a decline in one or more cognitive domains that goes beyond what expected from normal aging. The DSM-5 (1) distinguishes Mild-NCD (previously described as Mild Cognitive Impairment, MCI), characterized by a level of cognitive decline that requires compensatory strategies and accommodations to help maintain independence and perform activities of daily living, and Major-NCD (previously described as dementia), characterized by cognitive impairment which affects autonomy in activities of daily living. Despite the presence of cognitive disorders in central to the NCD definition, neuropsychiatric symptoms, such as apathy, depression, and anxiety, are also highly prevalent in both Mild- and Major-NCD (2). Specifically, apathy represents the most common behavioral symptom in NCD due to Alzheimer’s Disease (AD) and is often observed in Parkinson’s disease (PD), vascular dementia, stroke, traumatic brain injury, amyotrophic lateral sclerosis/motor neuron disease, frontotemporal dementia, progressive supranuclear palsy, and also in psychiatric conditions such as major depression and schizophrenia (3).

The definition and the diagnostic criteria for apathy (4–8) have evolved overtime, and the terminology employed to refer to apathy can vary in the context of different pathological condition. Today, apathy is considered as a clinical syndrome characterized by a reduction in self-initiated, goal-directed activity, which is not driven by primary motor or sensory impairments, or other co-morbidities such as drug intoxication or intercurrent illness (9). The apathy diagnostic criteria for NCD revised in 2021 identify three apathy domains, namely diminished initiative, diminished interests, and diminished emotional expression/responsiveness (10).

In the present paper, we focus on diminished interest, an apathy feature that has been identified also in the previous versions of the diagnostic criteria for apathy (6, 11), and is assessed in most of the classical clinical apathy scales, such as the NPI-Clinician rating scale (12), the Apathy Evaluation Scale (13), and the Apathy Inventory (14). In terms of prevalence, diminished interest is the most common dimension after reduced initiative, and is present in more than half of patients with Mild Neurocognitive disorders (15), alone or associated to other apathy symptoms (16).

To assess the presence of diminished interest, it is necessary ask the patient and/or the caregivers specific questions during the clinical interview. This can be done using the examples/questions provided in the diagnostic criteria, or using questions present in the apathy clinical scales such as the Lille Apathy Rating Scale (17), the Neuropsychiatric Inventory (18), the Apathy Inventory (14), the Dimensional Apathy Scale (19), and the Apathy Motivation Index (20). Self-report versions of scales can also be employed outside the clinical setting. Thanks to these instruments, clinicians can notify the presence of absence of diminished interest (0/1) and can quantify the symptom severity. In order to make this assessment more objective, and to guide the clinician during the interview, we developed a ludic application, named the “Interest game” (21). This application aims to propose a standardized procedure to collect systematically personal interests that can be used by clinicians, but also by the patient alone. Using this application, we showed that apathetic patients with neurocognitive disorders showed scores significantly lower than non-apathetic patients, and that the scores decreased from healthy controls to patients with Mild neurocognitive disorders to patients with Major neurocognitive disorders (21).

The aim of the present study is, first, to present results obtained on a bigger sample of participants (healthy controls, persons with Mild Neurocognitive Disorders, and persons with Major Neurocognitive disorders), and second to present cutoff scores for participants with Mild Neurocognitive disorders, a population with a high prevalence of lack of interest that can employ the application in autonomy for early screening. The definition of cutoff scores is crucial to promote the use of the application in a clinical setting.

## Methods

### Participants and procedures

The analyses were performed on 227 participants (83 males and 144 females, mean age = 75.9 years ±7.4) recruited at the Memory center of the University Hospital of Nice in the context of the MotAp (approved by the Comité de Protection de Personnes—CPP Est III, France; RCB ID No. 2017-A01366-4), Marcosens (Comité de Protection des Personnes—on 15/04/2019, RCB ID: 2019–A00342-55) and FAME-1 (approved by the Comité de Protection de Personnes—CPP Sud-Ouest et Outre Mer 1, France: RCB ID No.2020-A02025-34) studies. These included 56 healthy controls (HC), 118 persons with Mild-NCD, and 53 persons with Major-NCD based on the DSM-5 diagnostic criteria (1). Persons with NCD were not included if they had sensory or motor impairments interfering with the protocol completion. HC were recruited at the memory center among the patients’ caregivers and persons that came for a consultation but had no sign of cognitive impairment. A brief screening (including the MMSE) was performed to ascertain the absence of any cognitive decline. For the purposes of the present study, we extracted from the studies databases the following variables: demographics (age, sex, and level of education), diagnosis (HC, Mild-NCD and Major-NCD), severity of cognitive decline (as assessed by the Mini Mental State Examination, MMSE, (22)), presence of apathy (based on the Diagnostic Criteria for Apathy, DCA, (11)), apathy severity (when available, assessed with the apathy inventory, AI, (14)), and the results of the Interest game (number of categories and number of images selected, (21)).

### The Interest game

**Figure 1:**
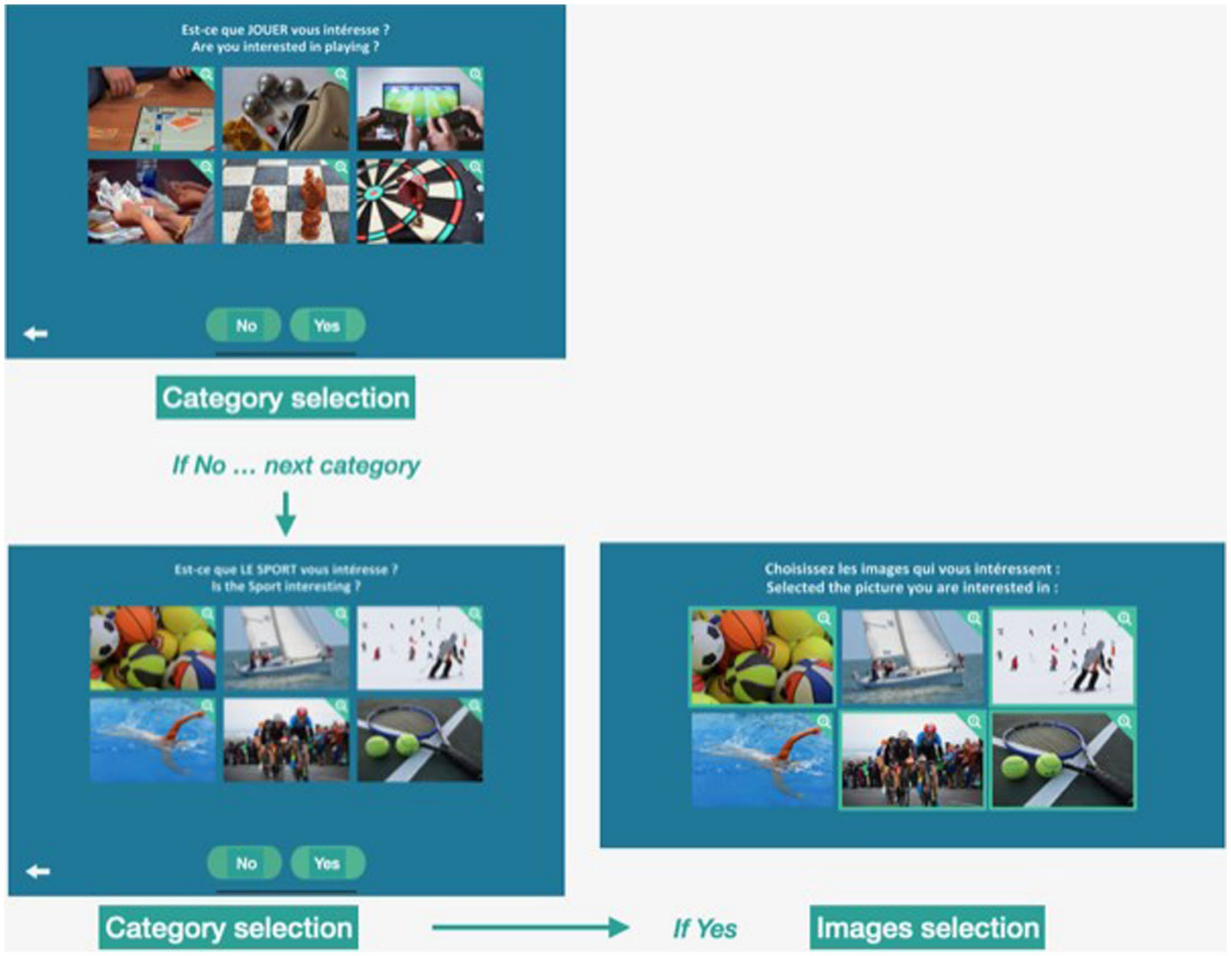
Interest game. Screenshots of the Interest game. First, participants are asked if they are interested in a specific category (e.g., playing). If they respond “No,” they go directly to the next category (e.g., sports). If they respond “Yes,” they are asked to select up to six images of activities they are interested in.

Starting from a survey aiming to define the most common interests in elderly people (23), 17 categories of interest were created: “Eating well,” “Singing,” “Dancing,” “Self-care,” “Playing,” “Family,” “The sea,” “The mountain,” “Nature,” “New technologies,” “Social interactions,” “Sports,” “Reading “Love,” “Museums and arts,” “Relaxation and meditation,” and “TV and cinema.” For each category, 6 images were selected representing different activities included in the same category (see Figure 1). For example, the sport category includes images of activities such as riding a bike, playing ball games, or sailing. For the “playing” category, different types of game are displayed as cards, video games, or bowling, etc. (see link for more information^1^). The Interest game has been included in the Motap application freely available on IOS and Android; Android^2^ and iOS^3^.

The game was carried out as follows: 1. A category is presented in the form of a question: Are you interested in “category” (example “sports”). 2. The subject answered “Yes” or “No.” 3. If the answer was “No,” subjects were presented with the next category of interest. If the answer was “Yes,” subjects were asked to select among 6 images those they are interested in. They could choose 0 to 6 images. Two scores were collected: (1) The total number of categories chosen (number of categories answered “yes”; Category). The maximum score was 17. (2) The total number of images selected for all the categories answered “yes” (Images). The maximum score was 102.

For the purposes of the study, the application was installed on a touchscreen tablet. The experimenter (a researcher with a clinical background) explained to the participants how to use the application, and how to select/unselect the responses (by touching the relevant responses/images). The experimenter clarified that the game is focused on personal interests, rather than asking if the person is currently performing the presented activity (a person can be interested in watching tennis on TV, even if he/she is does not play tennis). The experimenter stayed with the participants during the game, to respond to their questions, help them in case they needed (e.g., to go back on a previous screen to modify a response), and to ensure that they completed the task.

### Data analysis

Descriptive statistics were used to present demographic and clinical characteristics. Qualitative variables were presented using frequency and percentage, and quantitative variables were presented using mean and standard deviation (SD). For the clinical and demographic variables, Chi-square tests were employed to investigate differences in sex, level of education, and presence of the Apathy diagnostic criteria (ADC) among healthy controls and disorders categories (HC, persons with Mild-NCD and Major-NCD). One-way ANOVAs were performed to compare age, level of cognitive impairment (as indexed by the MMSE score) and degree of apathy (as indexed by the AI, total score and “interest” sub-score) among disorders categories. Postdoc paired-wise comparisons were performed applying Bonferroni correction. For the Interest game results, two types of analyses were conducted. First, in order to explore the factors affecting the Categories and Images scores, we performed a MANOVA with Categories and Images as dependent variables, disorders category, level of education, and presence of the ADC as factors, and Age and MMSE as covariate. *Post-hoc* paired-wise comparisons were performed applying Bonferroni correction. Pearson’s correlation analyses were also performed between results of the Interest game (Categories and Images) and AI (total score and “Interest” subscale). Second, we focused on persons with Mild-NCD (*N* = 118) and performed a ROC analysis to select cutoff scores on the Categories and Images scores based on the B1 dimension of the ADC. The AUC (Area Under the Curve) were indicated. These cutoffs were then employed to compute the sensitivity, specificity, and global accuracy of the Categories and Images scores in detecting the presence of lack of interest (B1 dimension of the ADC) in persons with Mild-NCD.

## Results

### Demographic and clinical data

The demographic and clinical features of the participants are summarized in Table 1. A significant effect of disorders on age (*F*_(2,224)_ = 6.9, *p* < 0.001) and MMSE (*F*_(2,224)_ = 70.5, *p* < 0.001) was found, with HC being significantly younger (*p* = 0.014) and with a higher MMSE (*p* < 0.001) than Mild-NCD and Major-NCD (*p* < 0.001) persons, and persons with Mild-NCD having a higher MMSE than persons with Major-NCD (*p* < 0.001). No significant effect of disorders on sex was found (*χ*^2^_(2,1)_ = 0.4, *p* = 0.828). Disorders showed a significant effect on the level of education (*χ*^2^_(2,2)_ = 15.9, *p* = 0.003), with HC showing a highest percentage of superior education (60.7%), and persons with Mild-NCD and Major-NCD showing a highest proportion of secondary education (46.6 and 49.1%, respectively). Also, disorders had a main effect on the presence of the ADC (*χ*^2^_(2,1)_ = 35.7, *p* < 0.001), with a percentage of persons meeting the ADC increasing from HC (8.9%) to persons with Mild-NCD (39.0%) and Major-NCD (64.2%). Similarly, disorders had a main effect on the presence of the B1-ADC subdomain (cognition/behavior) (*χ*^2^_(2,1)_ = 44.0, *p* < 0.001), with a percentage of persons positive to the B1 dimension increasing from HC (12.5%) to persons with Mild-NCD (47.5%) and Major-NCD (75.5%). Disorders had also a main effect on the AI (*F*_(2,224)_ = 23.4, *p* < 0.001) and AI-interest (*F*_(2,224)_ = 17.8, *p* < 0.001), with HC showing significantly lower AI and AI-interest scores compared to Mild-NCD and Major-NCD (all *p* < 0.001).

**Table 1:** Participants’ demographic and clinical features.

	Healthy controls (*N* = 56)		Mild NCD (*N* = 118)		Major NCD (*N* = 53)		
	Mean	[SD]	Mean	[SD]	Mean	[SD]	*p*-value*
Age	73.0	[7.3]	76.3	[7.3]	77.9	[6.7]	**<0.001**
MMSE	28.9	[1.4]	25.1	[3.1]	18.3	[3.9]	**<0.001**
Apathy inventory—total	0.3	[0.9]	2.2	[2.8]	3.1	[3.1]	**<0.001**
Apathy inventory—interests	0.2	[0.5]	0.9	[1.2]	1.2	[1.3]	**<0.001**
	n	(%)	n	(%)	n	(%)	*p*-value**
Sex							0.828
Female	22	(39.3)	41	(34.7)	20	(37.7)	
Male	34	(60.7)	77	(65.3)	33	(62.3)	
Level of education							**0.003**
Primary level	8	(14.3)	16	(13.6)	13	(24.5)	
Secondary level	14	(25.0)	55	(46.6)	26	(49.1)	
Superior level	34	(60.7)	47	(39.8)	14	(26.4)	
Apathy Diagnostic Criteria (ADC)								**<0.001**
No	51	(91.1)	72	(61.0)	19	(35.8)	
Yes	5	(8.9)	46	(39.0)	34	(64.2)	
ADC—B1 (Cognition/Behavior)							**<0.001**
No	49	(87.5)	62	(52.5)	13	(24.5)	
Yes	7	(12.5)	56	(47.5)	40	(75.5)	

*ANOVA; **χ^2^.

### Interest game

A multivariate analysis of the variance conducted on the results of the Interest game (Categories and Images) with disorders group (HC, Mild-NCD, Major-NCD), ADC (presence vs. absence), and Education level (Primary, Secondary, Superior) as between-subject factors and MMSE score and Age as covariates revealed a significant main effect of the ADC on both the number of selected Categories (*F*_(2,224)_ = 12.5, *p* < 0.001) and Images (*F*_(1,224)_ = 12.7, *p* < 0.001), with apathetic participants showing lower numbers of selected categories and images compared to non-apathetic participants (Supplementary Table 1). A significant effect of the Education level was also found on Categories (*F*_(2,207)_ = 3.9, *p* < 0.021) and an almost significant effect was found for the Images (*F*_(2,207)_ = 3.0, *p* = 0.052), with participants with primary education showing a significant lower number of selected categories compared to participants with superior education (*p* = 0.030). An effect trending toward significance was found also for disorders (*F*_(2,207)_ = 2.9, *p* = 0.059), with HC showing a higher number of selected Categories than Mild-NCD (*p* = 0.047) and Major-NCD (*p* = 0.015). No other significant main effects or interactions were found (all *p* > 0.05).

Correlation analyses revealed a moderate negative correlation between Categories and AI (AI-total, *r*_(164)_ = −0.42, *p* < 0.001; AI-Interest, *r*_(164)_ = −0.44, p < 0.001) and Images and AI (AI-total, *r*_(164)_ = −0.35, *p* < 0.001; AI-Interest, *r*_(164)_ = −0.35, *p* < 0.001), confirming that the number of selected categories and images decreases as apathy symptoms and reduced interest become more severe (Supplementary Table 2). Partial correlations (controlling for disorders category and Education level) confirmed the same results (all *p* < 0.01) (Supplementary Table 3).

### Cutoff definition

**Figure 2:**
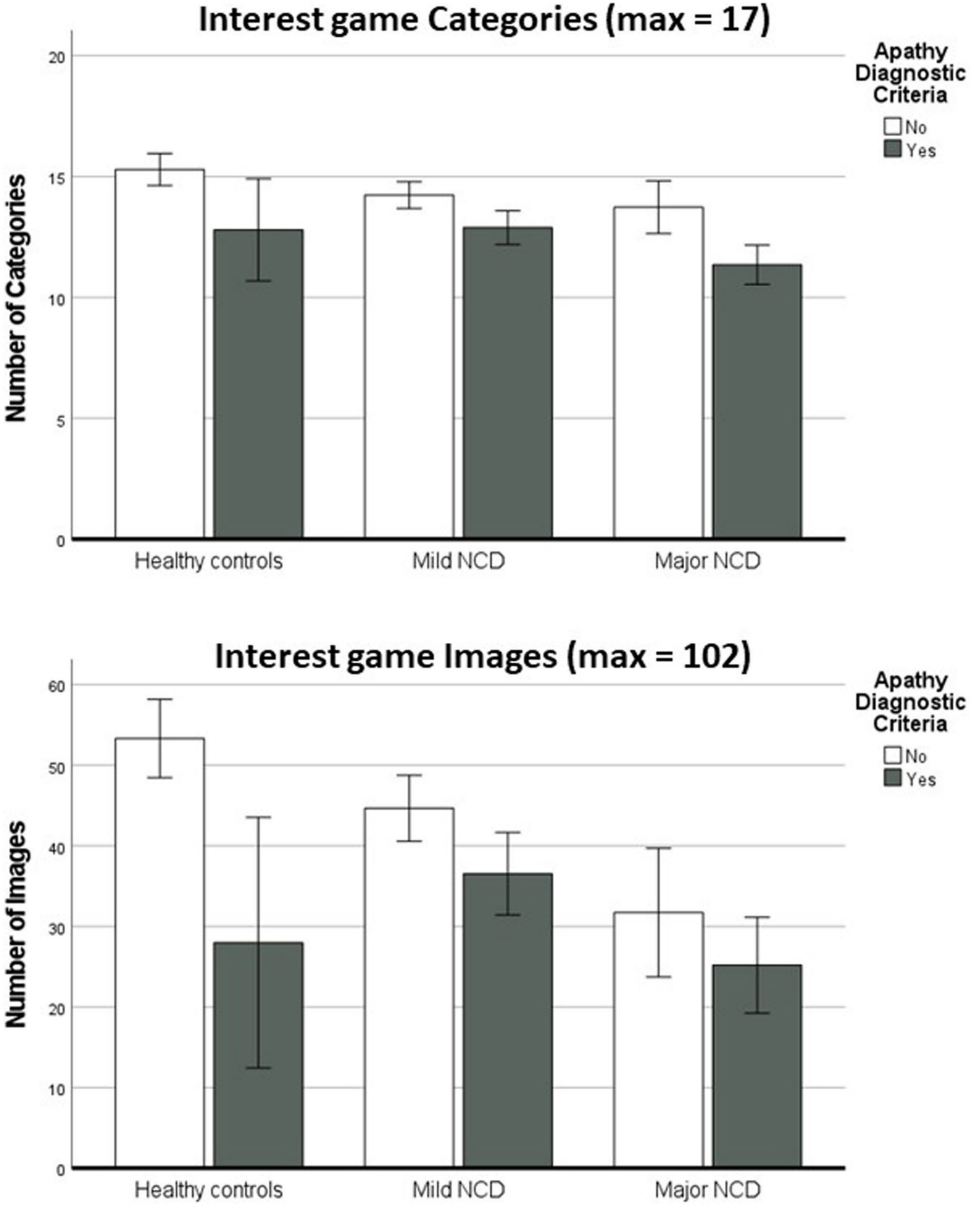
Interest game results (Categories and Images) for apathetic and non-apathetic participants in the three diagnostic groups Error bars represent ±2 standard errors.

In the present paper, we focused on the definition of cutoff scores for patients with Mild-NCD, for whom we have more than 100 participants (see Table 1). To establish cutoffs for the Categories and Images scores, we computed separate ROC curves using the B1 dimension of the ADC (Cognition/Behavior, (11)) as classification criterion (see Figure 2). This resulted in an AUC = 0.70 for the Category, and an AUC = 0.63 for the Images (see Supplementary Figure 1). We selected the cutoff scores in order to maximize the sensitivity (the probability to detect apathetic persons) without bringing specificity below 50% (the probability to detect non apathetic persons). This resulted in a cutoff score of 15 for the Categories (a score lower than 15 indicates the presence of diminished interest), and a cutoff score of 44 for the Images. The sensitivity, specificity, and accuracy for the Categories and Images are reported in Table 2.

**Table 2:** Sensitivity, specificity, and accuracy with the selected cutoff scores.

	Categories (cutoff <15)	Images (cutoff <44)
Sensitivity	68%	63%
Specificity	65%	52%

## Discussion

Diminished interest is a core feature of apathy in neurocognitive disorders (NCD) that manifests as reduced interest and enthusiasm in events happening in the environment, in activities and plans made by others, in friends and family, and reduced participation in activities even when stimulated (10, 11). In a recent survey performed in a clinical setting (16), we found that the frequency of apathy and diminished interest ranged from 25% in patients with mild-NCD, to 57% in patients with affective disorders (depression, anxiety, and bipolar disorders), to 77% in patients with major NCD. Diminished interest was also found in a substantial proportion of patients that did not meet the full spectrum of the Apathy Diagnostic Criteria, suggesting a very high prevalence in NCD. As apathy represents a risk factor for the conversion from Mild to Major-NCD (24, 25), early detection is of crucial importance, as it allows to put in place early and thus more effective treatment options (26).

Clinicians can rely on several reliable clinical scales to assess reduced interest in a consultation setting (11). However, fewer instruments do exist to be employed by patients alone, as self-report questionnaires suffer from several biases (e.g., presence of anosognosia, but also difficulty in understanding the questions and providing a reliable response employing rating scales). Here, we provided evidence on the utility of a ludic application, named “Interest game” for the assessment of diminished interest in elderly people with NCD.

Converging with previous findings (21), we found that the two indexes of the application (number of Categories and number of Images) were significantly lower in patients with the Apathy Diagnostic Criteria, even after controlling for the effects of disorder category, global level of cognitive impairment, age, and education, confirming the interest of employing the application for apathy assessment. Furthermore, significant correlations were found between Categories and Images and apathy severity (as indexed by the Apathy Inventory (14)), suggesting that the application scores converge with the results of clinical scales. Significant effects on the application scores were also found for the disorder category, with a progressive reduction of the number of selected Categories and Images from healthy controls to patients with Mild and Major-NCD. This is consistent with the literature and with the results of the clinical scales, confirming that apathy prevalence, including diminished interest, increases when the NCD severity increases (26, 27). Converging with our previous reports (21), we found that also the education level had an impact on the scores, with the Interest game scores decreasing with lower education levels. This is consistent with previous reports suggesting correlation between apathy scores and education (28, 29), and with the fact that higher education is a protective factor for dementia conversion (30).

To promote the use of the Interest game in the clinical practice, we calculated cutoff scores for the Categories and Images for patients with Mild-NCD, to provide references for clinicians to assess individual performance. Using a cutoff strictly lower than 15 for the Categories score, and strictly lower than 44 for the Images scores, we found a sensitivity of 68% and a specificity of 65% for the Categories, and a sensitivity of 63% and a specificity of 52% for the Images, suggesting that the Categories score discriminated slightly better than the Images score apathetic and non-apathetic NCD patients. Selecting higher cutoffs scores would have allowed to further increase sensitivity, but with the specificity and overall classification accuracy dropping lower the 50% level. The fact that the classification accuracy is not perfectly matched with the standard clinical assessment suggests that the Interest game, as any self-report instrument, suffers from possible biases (such as the awareness of the disorder), and thus should be used to complement classical clinical assessment and not to replace it. The self-report biases are especially important in people with NCD that can suffer from anosognosia (reduced awareness of the reduction of interest compared to the previous state) (31) and memory problems (reduced access to recent examples in which lack of interested was manifested) (32). Further studies including assessment of the presence of anosognosia and a precise quantification of episodic memory deficits should be performed to establish how these two conditions impact the application results. In addition, the presence of a reduced number of interests cannot be used, alone, to make a diagnosis of *diminished* interests, as patients may have had few interests also before, confirming the importance to assess the presence of diminished interest in a clinical setting.

In conclusion, despite some limitations, the Interest game may represent an easy-to-use additional tool for a general assessment of apathy in the elderly population, that is simple and fast to administer, and is minimally based on language. In a clinical setting, the Interest game is useful not only for the assessment, but also for orienting non-pharmacological treatment options. To be effective, apathy treatment should be adapted to the needs and interest of the patient (26). Collecting systematically the personal interest can allow to orient the patients more rapidly toward activities that match their preferences. Furthermore, a qualitative analysis of the selected images may provide valuable information on the patient’s environment and way of life, facilitating both assessment and treatment orientation.

As apathy and diminished interests can be found in other pathological conditions (e.g., schizophrenia, major depression, stroke, amyotrophic lateral sclerosis, (3)), it would be interesting to test the usability of the Interest game in these populations, and develop different versions of the application based on the participants’ age (as the interest categories may vary between adults and seniors), with age- and disorder- specific cutoff scores. Furthermore, it would be important to collect a bigger sample of healthy elderly participants recruited in the general population. Despite in our sample we controlled for the absence of cognitive impairment in the healthy control group, participants were recruited among caregivers and healthy elderly consulting the memory center. This represents a selection bias, as these participants show a higher prevalence of neuropsychiatric symptoms such as depression (33), which is also associated to diminished interest.

## Data availability statement

The raw data supporting the conclusions of this article will be made available by the authors, without undue reservation.

## Ethics statement

The studies involving human participants were reviewed and approved by Comité de Protection de Personnes—CPP Est III, France; RCB ID No. 2017-A01366-4. The patients/participants provided their written informed consent to participate in this study.

## Author contributions

VM and PR worked on the first draft of the manuscript. VM and RF performed the data analysis. LD, RZ, and MP collected the data. JL was in charge of the studies coordination. AG and GS wrote the study protocols. All authors contributed to the article and approved the submitted version.

## Funding

This work was supported by the Association IA, the JL Noisiez Fondation, and by the University Cote d’Azur, EUR Healthy, UCA JEDI grant number ANR-IS-IDEX-01.

## Conflict of interest

The authors declare that the research was conducted in the absence of any commercial or financial relationships that could be construed as a potential conflict of interest.

## Publisher’s note

All claims expressed in this article are solely those of the authors and do not necessarily represent those of their affiliated organizations, or those of the publisher, the editors and the reviewers. Any product that may be evaluated in this article, or claim that may be made by its manufacturer, is not guaranteed or endorsed by the publisher.

## Footnotes

^1^ http://www.innovation-alzheimer.fr/motivation-application-2/

^2^ https://bit.ly/motaps

^3^ https://bit.ly/motaps2
