# Supplementary figures and images for: Cutoff scores for the “Interest game”, an application for the assessment of diminished interest in neurocognitive disorders
Source: Front Psychiatry. 2023 Mar 20;14:1126479. doi: 10.3389/fpsyt.2023.1126479 (PMC10067876; doi:10.3389/fpsyt.2023.1126479)

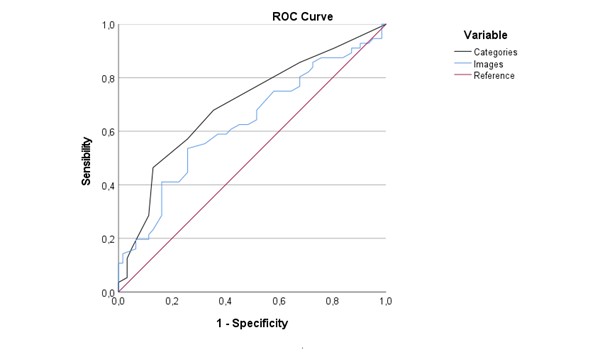

Supplement: Supplementary file 1 [file Image_1.JPEG]
