# Supplementary material for: Cutoff scores for the “Interest game”, an application for the assessment of diminished interest in neurocognitive disorders
Source: Front Psychiatry. 2023 Mar 20;14:1126479. doi: 10.3389/fpsyt.2023.1126479 (PMC10067876; doi:10.3389/fpsyt.2023.1126479)
Supplement: Supplementary file 2 [file Table_1.docx]

**Supplementary Table 1. MANOVA conducted on the results of the Interest game (Categories and Images) with disorders group (HC, Mild-NCD, Major-NCD), Apathy Diagnostic Criteria (presence vs absence), Education level (Primary, Secondary, Superior) as between subject factors and MMSE score and Age as covariates.**

|  | Dependent Variable | F | p | Partial Eta Squared |
| --- | --- | --- | --- | --- |
| MMSE | Interest game Categories | .68 | 0.409 | 0.00 |
|  | Interest game Pictures | 1.01 | 0.317 | 0.01 |
| Age | Interest game Categories | 2.44 | 0.120 | 0.01 |
|  | Interest game Pictures | .38 | 0.537 | 0.00 |
| Education | Interest game Categories | 3.94 | **0.021** | 0.04 |
|  | Interest game Pictures | 3.00 | 0.052 | 0.03 |
| Diagnosis | Interest game Categories | 2.87 | 0.059 | 0.03 |
|  | Interest game Pictures | 1.25 | 0.288 | 0.01 |
| ADC | Interest game Categories | 12.47 | **0.001** | 0.06 |
|  | Interest game Pictures | 12.65 | **<0.001** | 0.06 |
| Education * Diagnosis | Interest game Categories | 1.90 | 0.112 | 0.04 |
|  | Interest game Pictures | 1.68 | 0.155 | 0.03 |
| Education * ADC | Interest game Categories | .50 | 0.606 | 0.01 |
|  | Interest game Pictures | .25 | 0.779 | 0.00 |
| Diagnosis * ADC | Interest game Categories | .91 | 0.403 | 0.01 |
|  | Interest game Pictures | 2.70 | 0.070 | 0.03 |
| Education * Diagnosis * ADC | Interest game Categories | 1.13 | 0.337 | 0.02 |
|  | Interest game Pictures | 1.12 | 0.341 | 0.02 |

**Supplementary Table 2. Correlations between the Interest game and the Apathy Inventory**

|  | | | | | |
| --- | --- | --- | --- | --- | --- |
|  | | Interest game Categories | Interest game Images | Apathy Inventory | Apathy Inventory B1 |
| Interest game Categories | Pearson Correl | 1 | 0.63^**^ | -0.42^**^ | -0.44^**^ |
|  | Sig. (2-tailed) |  | <0.001 | <0.001 | <0.001 |
|  | N | 227 | 227 | 166 | 166 |
| Interest game Images | Pearson Correl | 0.63^**^ | 1 | -0.34^**^ | -0.35^**^ |
|  | Sig. (2-tailed) | 0.000 |  | <0.001 | <0.001 |
|  | N | 227 | 227 | 166 | 166 |
| Apathy Inventory | Pearson Correl | -0.42^**^ | -0.34^**^ | 1 | 0.90^**^ |
|  | Sig. (2-tailed) | 0.000 | 0.000 |  | 0.000 |
|  | N | 166 | 166 | 166 | 166 |
| Apathy Inventory B1 | Pearson Correl | -0.44^**^ | -0.35^**^ | 0.90^**^ | 1 |
|  | Sig. (2-tailed) | 0.000 | 0.000 | 0.000 |  |
|  | N | 166 | 166 | 166 | 166 |
| **. Correlation is significant at the 0.01 level (2-tailed). | | | | | |

**Supplementary Table 3. Partial correlations between the Interest game and the Apathy Inventory, corrected by Diagnosis group and Education level**

|  | | | | | | |  |
| --- | --- | --- | --- | --- | --- | --- | --- |
| Control  Variables | | | Interest game Categories | Interest game Images | Apathy Inventory | Apathy Inventory B1 |  |
| Diagnosis, Education | Interest game Categories | Correlation | 1.000 | 0.58** | -0.33** | -0.35** |  |
|  |  | Sig (2-tailed) | . | <0.001 | <0.001 | <0.001 |  |
|  |  | df | 0 | 162 | 162 | 162 |  |
|  | Interest game Images | Correlation | 0.58** | 1.000 | -0.22** | -0.24** |  |
|  |  | Sig (2-tailed) | <0.001 | . | 0.005 | 0.002 |  |
|  |  | df | 162 | 0 | 162 | 162 |  |
|  | Apathy Inventory | Correlation | -0.325** | -0.22** | 1.000 | 0.89** |  |
|  |  | Sig (2-tailed) | <0.001 | 0.005 | . | <0.001 |  |
|  |  | df | 162 | 162 | 0 | 162 |  |
|  | Apathy Inventory B1 | Correlation | -0.35** | -0.24** | 0.89** | 1.000 |  |
|  |  | Sig (2-tailed) | <0.001 | 0.002 | <0.001 | . |  |
|  |  | df | 162 | 162 | 162 | 0 |  |
| **. Correlation is significant at the 0.01 level (2-tailed). | | | | | | | |
